# Triphallia (triple penis), the first reported case in human

**DOI:** 10.1016/j.ijscr.2020.11.008

**Published:** 2020-11-04

**Authors:** Shakir Saleem Jabali, Ayad Ahmad Mohammed

**Affiliations:** Department of Surgery, College of Medicine, University of Duhok, Kurdistan Region, Iraq

**Keywords:** Triphallia, Diphallia, Supernumerary penises, Multiple penises, Urogenital anomalies

## Abstract

**Introduction:**

Supernumerary penises is an extremely rare congenital anomaly which affects one in every 5–6 million live births. Affected patients may have only a rudimentary penis, supernumerary penile glances or complete duplication or triplication of penises. Some patients may have some other associated congenital anomalies.

**Presentation of case:**

A 3-month-old child presented because of left side hydrocele. There were evidence of two supernumerary penises in the perineum, the first one was about 2 cm in length with a glans and was attached to the root of the penis, and the third one was about 1 cm and was below the scrotum. Hydrocelectomy was performed. The two supernumerary penises were extending to perineal region and were attached to original penis, both had corpora cavernosum and spongiosum with no urethra inside. Both supernumerary penises were excised and both corpora were sutured with a fine slowly absorbable suture material. The patient was discharged with no postoperative events and follow up was done for one years with no reported adverse events.

**Conclusion:**

Triphallia (three penises) is unreported condition in human until now. Patients with supernumerary penises have unique presentation and no cases are identical. The position of the penis may be ectopic or orthotopic. Treatment is difficult because it poses medical, ethical, and cosmetic aspects. A combined multidisciplinary team is required for the management and long term follow up is required. Excision or reconstruction of the duplicate penis is required depending on the corporal development and anatomy of the urethra.

## Highlights

• Supernumerary penises is an extremely rare congenital anomaly.
• It affects one in every 5–6 million live births.
• Each case has a unique presentation and no cases are identical.
• Around 100 cases of diphallia are reported till now.
• No cases of Triphallia is reported before.

## 1 Introduction

Supernumerary penises is an extremely rare congenital urogenital anomaly which was first reported in 1609 by Wecker, after that around 100 cases of diphallia are reported in literature. Duplication of the penis or diphallia is reported to affect one in every 5–6 million live births. The extent of this anomaly and other associated anomalies vary greatly from patient to another. Affected patients may have only a rudimentary penis, supernumerary penile glances or complete duplication or triplication of penises. Some patients may have some other associated congenital anomalies [[1], [2], [3]].

This condition occurs during the embryonic development of the penis between the 3rd and the 6th weeks of gestation, or may occur during the process of ventral migration and fusion of the paired mesodermal anlagen at the 15th week of gestation. Strong environmental factors like drugs and infections may affect the fetal caudal mass of mesoderm during this period suggesting that epigenetic mechanism may play a role in pathogenesis of diphallia [1,3].

Penile duplication is divided on the basis of the presence of one or two corpora cavernosa in each penis into bifid phallus and true diphallia respectively, the term pseudo-diphallia is used to describe a small rudimentary penis. Most cases have a single corpus cavernosum in each organ; i.e. bifid phallus [1,4].

The penile urethra may show a wide range of associated anomalies, some patients may have a completely functioning urethra with normally placed meatus while others may have complete absent urethra [1].

Imaging particularly ultrasound is helpful to confirm the diagnosis by detecting the presence or absence of corpora cavernosum or corpora spongiosum and their number, it also may detect other associated anomalies, magnetic resonance imaging provide a better quality of images in delineating the anatomy which is helpful to make an appropriate surgical decision [1,5].

The treatment of this condition is difficult because it poses medical, ethical, and cosmetic aspects. A combined multidisciplinary team is usually required for the management and long term follow up is usually required [1,6].

To the best of our knowledge, this is the first reported case with three penises or triphallia as no similar case is present in literature in human beings. This will add to literature because this may change the classification of multiple penises and help the authors for standardizing the management planes according to individual cases.

The work of this report case has been reported in line with the SCARE 2018 criteria [7].

## 2 Patient information

A 3-month-old child, who is native Kurd from Duhok city, presented by his parents because of left sided scrotal swelling and 2 skin projections in the perineum.

There was negative history for drug exposure during pregnancy. The family history for any relevant genetic abnormalities and psychosocial histories were also negative.

### 2.1 Clinical findings

The general examination revealed no abnormal findings.

**Fig. 1:**
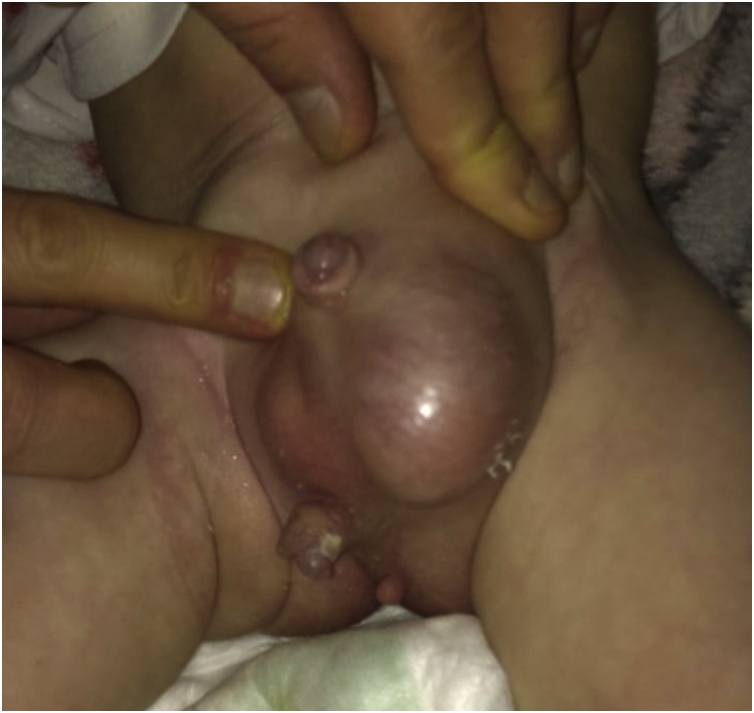
A preoperative picture showing the original and the two supernumerary penises.

Examination of the genitalia revealed evince of a left sided scrotal swelling which was trans-illuminating on applying light suggesting a hydrocele. There were evidence of two projections in the perineum, the first one was about 2 cm in length with glans and was attached to the root of the penis, and the third one was about 1 cm and was below the scrotum, the two projections were consistent with supernumerary penises Fig. 1.

### 2.2 Diagnostic assessment

Ultrasound examination of the abdomen, kidneys, ureters, and urinary bladder showed no other associated anomalies.

### 2.3 Therapeutic intervention

Surgery was performed under general anesthesia, during surgery the hydrocele sac (the patent processus vaginalis) was ligated at the inguinal canal.

The two supernumerary penises were extending up to perineal region and were attached to original penis, both had corpora cavernosum and spongiosum with no urethra inside. Both supernumerary penises were excised and both corpora were sutured with a fine slowly absorbable suture material, the skin was then sutured with fine interrupted suture material.

The operation was performed by a consultant urologist who is expert in the field of uro-surgery and penile reconstruction.

### 2.4 Follow-up and outcomes

The patient was discharged with no postoperative events and follow up was done for one years with no reported adverse events.

The family were advised about regular visits especially at the time of puberty and before marriage.

## 3 Discussion

The condition of supernumerary penises is an extremely rare congenital anomaly and each case has a unique presentation and no cases are identical. The position of the penis may be ectopic or orthotopic [1,3].

As the karyotype is normal (46XY) in most cases, this make it difficult to explain the exact cause for the duplication because the genital tubercle is not a paired structure during normal embryonic development. Many theories are present suggesting its pathogenesis, but there is still a great debate and none of them is convincing [4].

Many genetic alterations have been implicated in the development of supernumerary penises, some of these include genes which encode for the expression of androgen receptors and are linked to the development of the male external genitalia [3].

There are numerous associated anomalies which are reported in such patients, these may include abnormalities of the meatus like hypospadius, or epispadius, anomalies of the scrotum like bifid scrotum, anomalies of the testes like ectopic or undescended testis, anomalies of the blabber or the lower gastrointestinal tract, skeletal or pelvic bone anomalies, or cardiac anomalies. When the infant has major associated congenital anomalies, it is associated with high death rates [1,[8], [9], [10]].

Many clinical classifications are present for this conditions, some authors classify it into a true supernumerary penis or just a bifid penis, which is further subdivided into partial or complete. The true complete type refers to complete duplication in which each penis has two corpora cavernosa and one corpus spongiosum, the partial type include either smaller or rudimentary penis. The term bifid phallus is applied when each penis has only one corpus cavernosum. In other cases only the glans is duplicated [1,4].

The extent of erectile function in such cases varies greatly, one or both penises may achieve erection, and rarely simultaneous ejaculation is reported if the condition is left untreated until puberty, while in pseudo-diphallia this is not present due to rudimentary phallus [1].

There is no standard managing plan for all cases and the choice of the treatment plan depends greatly on the presence or absence of type of other associated congenital anomalies and the preservation of the continence and the erectile function. The aim of surgical intervention should be good urinary continence and stream, proper erection and an adequate cosmetic appearance [1].

As this case is the first reported case, the management was based on the published papers, as many similarities are present between the cases. The rationale behind our surgical approach was that one of the supernumerary penises was apparently normal with urethra and the other 2 were accessary penises which were lacking urethra.

The surgical procedure is individualized for each patient as each case has a unique presentation and may be associated with many other congenital anomalies. Surgery usually include excision of the supernumerary non-communicating penis or penises, penile reconstruction by joining both corporal bodies of each penis is recommended in cases of true complete diphallia, and urethral reconstruction [1,4].

## 4 Conclusion

Triphallia (three penises) is unreported condition in human until now. Patients with supernumerary penises have unique presentation and no cases are identical. The position of the penis may be ectopic or orthotopic. Treatment is difficult because it poses medical, ethical, and cosmetic aspects. A combined multidisciplinary team is required for the management and long term follow up is required. Excision or reconstruction of the duplicate penis is required depending on the corporal development and anatomy of the urethra.

## Declaration of Competing Interest

The author has no conflicts of interest to declare.

## Funding

None.

## Ethical approval

Ethical approval has been exempted by my institution for reporting this case.

## Consent

An informed written consent was taken from the family for reporting the case and the accompanying images.

## Author’s contribution

Dr Shakir Saleem Jabali and Dr Ayad Ahmad Mohammed contributed to the concept of reporting the case and the patient data recording.

Drafting the work, design, and revision done by Dr Ayad Ahmad Mohammed.

Final approval of the work to be published was done by Dr Ayad Ahmad Mohammed and Dr Shakir Saleem Jabali.

## Registration of research studies

N/A.

## Guarantor

Dr Ayad Ahmad Mohammed is guarantor for the work.

## Provenance and peer review

Not commissioned, externally peer-reviewed.

## Patient perspective

The family was worried about the future sexual life of their child, and they were informed that long term follow up is required especially at the time of puberty and before marriage.
